# New developments in chondrocyte ER stress and related diseases

**DOI:** 10.12688/f1000research.22275.1

**Published:** 2020-04-24

**Authors:** Michael D. Briggs, Ella P. Dennis, Helen F. Dietmar, Katarzyna A. Pirog

**Affiliations:** 1 Biosciences Institute, Faculty of Medical Sciences, Newcastle University, International Centre for Life, Central Parkway, Newcastle upon Tyne, NE1 3BZ, UK

**Keywords:** cartilage, chondrocyte, skeletal dysplasia, endoplasmic reticulum, osteoarthritis, drug repurposing

## Abstract

Cartilage comprises a single cell type, the chondrocyte, embedded in a highly complex extracellular matrix. Disruption to the cartilage growth plate leads to reduced bone growth and results in a clinically diverse group of conditions known as genetic skeletal diseases (GSDs). Similarly, long-term degradation of articular cartilage can lead to osteoarthritis (OA), a disease characterised by joint pain and stiffness. As professionally secreting cells, chondrocytes are particularly susceptible to endoplasmic reticulum (ER) stress and this has been identified as a core disease mechanism in a group of clinically and pathologically related GSDs. If unresolved, ER stress can lead to chondrocyte cell death. Recent interest has focused on ER stress as a druggable target for GSDs and this has led to the first clinical trial for a GSD by repurposing an antiepileptic drug. Interestingly, ER stress markers have also been associated with OA in multiple cell and animal models and there is increasing interest in it as a possible therapeutic target for treatment. In summary, chondrocyte ER stress has been identified as a core disease mechanism in GSDs and as a contributory factor in OA. Thus, chondrocyte ER stress is a unifying factor for both common and rare cartilage-related diseases and holds promise as a novel therapeutic target.

## Introduction

Cartilage comprises a single cell type, the chondrocyte, embedded in a highly complex and macromolecule-rich extracellular matrix (ECM). Chondrocytes are a quintessential example of a ‘professional secretory cell’ and are particularly susceptible to homeostasis in endoplasmic reticulum (ER) protein-folding homeostasis.

**Figure 1.**
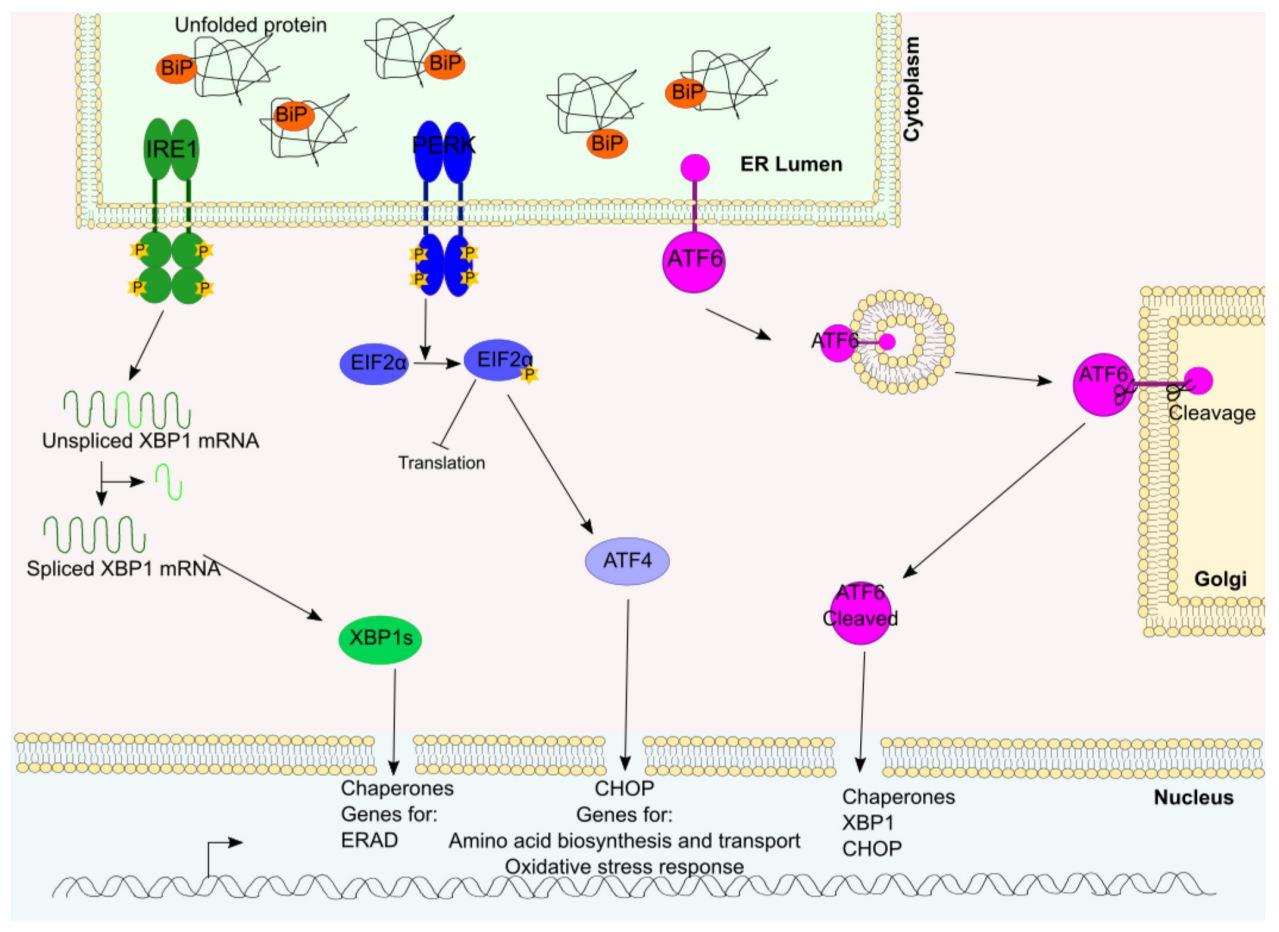
The unfolded protein response (UPR) is activated by ER stress and works through three distinct pathways. The accumulation of misfolded proteins within the endoplasmic reticulum (ER) lumen initiates the UPR. Misfolded proteins are recognised by binding immunoglobulin protein (BiP), which dissociates from three transmembrane-bound receptors—protein kinase ribonucleic acid-like endoplasmic reticulum kinase (PERK), inositol-requiring enzyme 1 (IRE-1) and activating transcription factor 6 (ATF6)—and binds to the exposed hydrophobic residues on the unfolded protein. Following BiP dissociation from PERK, it homodimerises, leading to autophosphorylation. PERK phosphorylates and inactivates the alpha subunit of eukaryotic translation initiation factor 2A (eIF2α) required for mRNA 5′ capping and 80s ribosomal activity. This leads to the reduction of global protein synthesis, thus reducing the load on the ER. BiP dissociation from the luminal domain of IRE-1α leads to homodimerisation and activation of its ribonuclease activity. Active IRE-1 cleaves a 26-nucleotide intron sequence from unspliced XBP1 (XBP1u) messenger RNA, resulting in the translation of spliced XBP1 (XBP1s). Spliced XBP1 functions as a transcription factor and migrates to the nucleus, where it binds the promoters and up-regulates genes encoding chaperones, protein disulphide-isomerases (PDIs) and genes encoding the proteins of ER-associated protein degradation. ATF6 consists of an ER luminal domain that binds BIP and a cytoplasmic domain of a bZip transcription factor. Dissociation of BiP from ATF6 allows for interactions between ATF6 and the COPII vesicular complex. ATF6 translocates to the Golgi apparatus, where it undergoes cleavage by site 1 and site 2 proteases. Following cleavage, the cytosolic portion of ATF6 acts as a transcription factor, binding to the promoters of target genes encoding chaperones and genes involved in ER-associated protein degradation and apoptosis. Therefore, these three signalling pathways are activated with the ultimate aims of ceasing translation, degrading the misfolded protein and (if the stress cannot be overcome) promoting cell death.

The ER is the largest organelle within a cell and functions as the site of protein synthesis and folding and as the start of the secretory pathway. ER stress occurs when the ability of the ER to fold nascent proteins becomes compromised and can result from a number of different factors, including viral infection, hypoxia, protein overexpression, the expression of mutated polypeptides that remain misfolded and deficiencies in protein folding, trafficking and degradation components and pathways. In order to detect and respond efficiently to ER stress, eukaryotic cells possess an evolutionarily conserved signal transduction cascade known as the unfolded protein response (UPR) ^1^. The UPR is initiated by the ER lumen resident chaperone glucose-regulated protein 78/binding immunoglobulin protein (GRP78/BiP) dissociating from three transmembrane-bound receptors—protein kinase RNA-like ER kinase (PERK), inositol-requiring enzyme 1 (IRE-1) and activating transcription factor 6 (ATF6)—and binding to the exposed hydrophobic residues on the unfolded protein. This results in the activation of three distinct receptor signalling pathways with the ultimate aims of decreasing protein translation and degrading the misfolded protein ( Figure 1). IRE-1 is an endonuclease and a trans-autophosphorylation kinase that in an active form removes an intron from the X-box binding protein 1 (XBP1) mRNA, allowing it to become a functional transcription factor (XBP1s). XBP1s then upregulates the expression of ER chaperone genes and genes involved in ER-associated protein degradation (ERAD) that facilitate recovery from ER stress. Upon activation, ATF6 translocates to the Golgi apparatus, where it is cleaved into an active form that acts as a transcription factor, further upregulating a subset of ER chaperone and ERAD genes either independently or in conjunction with Xbp1s. ATF6 can also co-signal with the final branch of the UPR, the PERK pathway. Activation of PERK leads to phosphorylation of a pro-survival elongation factor eukaryotic translation initiation factor 2A (eIF2α) that results in attenuation of protein translation. However, several genes, including ATF4 and C/EBP homologous protein (CHOP), that are downstream effectors of the PERK pathway can escape that attenuation. If the stress on the ER is not returned to physiological levels, the UPR activates a pro-apoptotic signalling cascade by inducing (CHOP)-dependent/-independent cascades ^2, 3^. This can have a detrimental effect on the chondrocyte phenotype, including reduced proliferation in the growth plate of long bones and eventually leading to cell death. Moreover, ER stress forms part of the integrated stress response ^4^ and forms part of the stress crosstalk together with inflammation, oxidative stress and autophagy ^5– 8^.

The induction of ER stress and the UPR as a core disease mechanism has been described in a number of phenotypically different rare diseases, including genetic skeletal diseases (GSDs), cardiovascular diseases and inherited myopathies, and is also implicated in more common conditions such as cancer and osteoarthritis (OA). Moreover, ER stress has been identified as a possible therapeutic target in a broad group of human diseases. The focus of this review is to provide an update on recent advances in chondrocyte ER stress in relation to GSDs and OA.

## Chondrocyte endoplasmic reticulum stress in rare bone diseases and during skeletal development

### Endoplasmic reticulum stress in rare genetic skeletal diseases due to misfolding of extracellular matrix proteins: recent advances in potential therapies

An extremely complex and diverse group of diseases, GSDs affect primarily the homeostasis and development of the skeleton. The severity of the more than 450 unique and well-characterised phenotypes range from relatively mild to severe and lethal forms. Individually, GSDs are rare but as a group of related orphan diseases they have an overall prevalence of at least 1 per 4000 children, representing a considerable unmet medical need ^9^.

Over the last decade, ER stress has been identified as a core disease mechanism in a group of phenotypically unrelated GSDs, all of which result from dominant-negative mutations in a range of cartilage ECM structural proteins, particularly those GSDs resulting from mutations in the genes encoding cartilage oligomeric matrix protein (COMP), matrilin-3, and collagen types II and X ^10^.

Archetypical examples of GSDs resulting from the accumulation of mutant cartilage proteins in the ER were first described several decades ago and include pseudoachondroplasia (PSACH), multiple epiphyseal dysplasia (MED) and metaphyseal chondrodysplasia type Schmid (MCDS) ^11^. More recently, our understanding of genotype-specific differences in ER stress pathways and the activation of different branches of the UPR has provided potential avenues for novel therapies. This is best exemplified by MCDS that is caused by missense or nonsense mutations in the gene encoding type X collagen ( *COL10A1*).

***Type X collagen and metaphyseal chondrodysplasia type Schmid.*** A study by Mullan *et al*. elegantly demonstrated that ER stress and the resulting growth plate pathology and reduced bone growth induced through the ER accumulation of mutant type X collagen can be corrected by the administration of carbamazepine (CBZ) in both cell and mouse models ^12^. Moreover, that study confirms that the mode of action of CBZ is to enhance mutant protein degradation by either the proteasome or autophagy in a mutation-dependent manner. In a follow-up study, it was demonstrated that an MCDS nonsense mutation in *Col10a1* also caused mutant collagen X retention, ER stress and an UPR in hypertrophic chondrocytes ^13^. Moreover, treatment of this mouse model of MCDS with CBZ reduced the disease severity in a manner similar to that previously described ^12, 13^. Overall, these complementary studies provide confidence that CBZ is likely to be effective in an on-going clinical trial for patients with MCDS in which the disease is caused by nonsense or missense *COL10A1* mutations (see https://mcds-therapy.eu).

Interestingly, whilst *Xbp1* was alternatively spliced in the MCDS mouse models ^14, 15^, the predominantly active pathways were the ATF6 and PERK branches of the UPR. Therefore, the role of the AFT6 branch of the UPR in relation to ER stress caused by the expression of mutant type X collagen has been studied in detail using cell and mouse models ^16^. Previously, ATF6β has been overlooked as having little or no functional significance and is redundant to ATF6α. However, studies by Forouhan *et al*. confirmed that both isoforms have distinct functions under ER stress. Moreover, this study identified that the disease severity of MCDS is modulated by the opposing roles of ATF6α (chondroprotective) and ATF6β (detrimental). This brings a level of complexity to the ATF6 branch of the UPR, highlighting the functional significance of ATF6β under ER stress conditions and therefore outlining its possible therapeutic potential, which has been ignored in many diseases associated with ER stress.

Interestingly, it has been suggested that ATF4 expression (downstream of PERK) in the MCDS mouse model may in fact be pro-survival and involved in the de-differentiation pathways observed in the *Col10a1* mutant chondrocytes. Indeed, ATF4 appeared to be upregulated in differentiating, proliferative chondrocytes, indicating zonal or differentiation state differences in the cartilage UPR responses. Overexpression of ATF4 in hypertrophic chondrocytes leads to a chondrodysplasia through reprogramming of hypertrophic cells towards *Sox9* expression and a more juvenile phenotype ^15^. Moreover, treating MCDS mice with the integrated stress response inhibitor (ISRIB) compound alleviates the MCDS phenotype ^15^. ISRIB renders cells insensitive to eIF2α phosphorylation without affecting the phosphorylation process itself ^17, 18^. Interestingly, treatment of MCDS mice with ISRIB restores bone growth and ablates the preferential translation of *Atf4* and *Chop* whilst the *Xbp1s* signals remain unaffected.

***Matrilin-3 and multiple epiphyseal dysplasia.*** Similar to MCDS, those forms of MED caused by *MATN3* mutations ( *MATN3*-MED) have ER stress and classic UPR as a core disease mechanism ^19^; however, unlike MCDS, cell and mouse models of *MATN3*-MED do not respond to CBZ treatment (unpublished data). This dichotomy in drug response might be best explained by a recent study by Pirog *et al*., who demonstrated that different pathways of the UPR are activated depending on the differentiation state of growth plate chondrocytes ^20^. For example, growth plate pathology is most pronounced in the proliferative zone of the *MATN3*-MED mouse model, in contrast to the restricted hypertrophic zone pathology in both MCDS mouse models, and deletion of *Xbp1* impacts on chondrocyte proliferation but not survival ^14^. However, in both MCDS and *MATN3*-MED, the XBP1 branch of UPR is activated and there are clear similarities in gene expression changes. Therefore, it was unexpected that the genetic deletion of *Xbp1* signalling in an MCDS mouse model resulted in no overt change in phenotype ^21^ but that, in contrast, it caused a significant worsening of the phenotype in an *MATN3*-MED mouse model ^20^. Therefore, the authors concluded that the differentiation state of chondrocytes within the cartilage growth plate influences UPR signalling and that these differences could inform future therapies.

***Cartilage oligomeric matrix protein and pseudoachondroplasia.*** ER stress is one of the stress pathways that form a network of interactions, together with oxidative stress, inflammation and autophagy. PSACH is another classic ER stress–related disease ^10^; however, numerous previous studies using both *in vitro* ^22, 23^ and *in vivo* ^24, 25^ models have unequivocally demonstrated that ER retention of mutant COMP induces ER overload response (EROL) ^26^ that comprises oxidative stress and inflammation potentially mediated by nuclear factor kappa B (NF-κB) as opposed to a ‘classic’ UPR ^10^. This observation identified new avenues for therapeutic intervention, including anti-inflammatory/antioxidant compounds such as aspirin and resveratrol ^27^. More recently, a role for mammalian target of rapamycin (mTOR) complex 1 in the pathogenesis of PSACH was demonstrated and suggested as a therapeutic target for COMPopathies ^28^.

### Chondrocyte endoplasmic reticulum stress resulting from disrupted protein folding and trafficking

The correct structure and function of cartilage require the coordinated synthesis and secretion of a plethora of ECM components, including a broad range of macromolecules such as collagens, glycoproteins and proteoglycans. Chondrocytes, as professionally secreting cells, are particularly susceptible to ER stress and therefore the UPR plays a vital protective role. In addition to the genetic mutations described in the previous section, any disruption to normal protein folding or deletion of individual components of the UPR can have a profound detrimental effect. This has recently been illustrated by the engineered deletion in mouse chondrocytes of two important components of the protein-folding machine—mesencephalic astrocyte-derived neurotrophic factor (MANF) ^29^ and ER resident protein 57 (ERp57) ^30^—whereas compound heterozygosity for mutations in site 1 protease (S1P) ^31^, a Golgi resident protease, causes a human chondrodysplasia.

***Mesencephalic astrocyte-derived neurotrophic factor.*** MANF is an ER resident protein that is increased and secreted during ER stress due to an imperfect KDEL sequence. The global knockout of MANF (on C57BL/6) is lethal at birth because mice die of respiratory distress due to decreased saccular formation in the lungs ^29^. Interestingly, however, crossing MANF ^−/−^ mice onto a Sv129 background alleviates the phenotype to the extent that mice survive to about 6 to 9 weeks of age. The cartilage-specific knockout of MANF is viable but causes a severe chondrodysplasia characterised by reduced long-bone lengths, confirming that endochondral ossification was disrupted ^29^. Despite morphologically normal growth plates, there was a concurrent reduction in chondrocyte proliferation in the cartilage-specific MANF null mice. This reduction in long-bone growth can be explained by the RNA sequencing (RNA-seq) analysis of chondrocytes from global and cartilage-specific knockout mice, which demonstrated increased expression of specific ER stress–related genes and a dysregulation of genes associated with chondrocyte proliferation, NF-κB signalling and terminal differentiation of chondrocytes.

***Protein disulphide-isomerase A3/endoplasmic reticulum resident protein 57 (PDIA3/ERp57).*** The cartilage-specific knockout of ERp57 has previously been shown to cause ER stress and UPR, resulting in reduced chondrocyte proliferation and dysregulated apoptosis leading to abnormal bone growth ^30^. More recently, the administration of 4-phenylbutyric acid (4-PBA), a small chemical chaperone, to ERp57-deficient chondrocytes in micromass or explant cultures reduced ER stress ^32^. Although these studies suggest that 4-PBA can alleviate ER stress *in vitro*, how effective it will be *in vivo* remains to be determined; indeed, previous studies in *MATN3*-MED mice have suggested limited efficacy ^19^.

***Site 1 protease.*** Mutations in S1P, a serine protease ubiquitously present in the Golgi apparatus and encoded by the *MBTPS1* gene, cause a novel human recessive skeletal dysplasia ^31^. Significantly, the mutations cause an almost complete loss of *MBTPS1* mRNA transcripts that disrupts ER function, particularly in chondrocytes. More precisely, reduced levels of S1P protein impair the activation of the ER stress response gene *BBF2H7*, leading to the retention in the ER of type II collagen. This ultimately causes prolonged ER stress, resulting in increased chondrocyte apoptosis. Interestingly, the correction of the *MBTPS1* mutations through antisense morpholino oligonucleotides or by the administration of PBA reduced collagen retention in the ER, suggesting a potential therapeutic avenue for this class of ER stress diseases.

## Chondrocyte endoplasmic reticulum stress in osteoarthritis

OA is a degenerative disease affecting multiple tissues in the diarthrodial joints and the spine. Risk factors in OA progression include age, sex, trauma, body weight, epigenetic modifications and genetic susceptibility ^33, 34^. OA progression is a complex process comprising cell senescence, developmental reprogramming, inflammation, catabolic/anabolic imbalance and ECM modifications.

### Endoplasmic reticulum stress markers in osteoarthritis

Interestingly, the levels of ER stress markers BiP ^35^, CHOP and caspase-12 correlate and increase with OA severity ^36^, and OA-associated processes, such as biomechanical injury, age-related glycation, chronic inflammation, and oxidative stress, have been shown to trigger UPR responses in several studies ^37– 41^. CHOP ^36, 42, 43^, alternatively spliced XBP1 ^39, 44^ and BiP ^35, 45^ have been shown to be increased in multiple *in vitro* and *in vivo* OA studies, and it has been suggested that CHOP-mediated apoptosis is an important component of cartilage degeneration during OA progression. Moreover, interleukin 1 (IL-1) and IL-6 are important cytokine components in OA progression, and ER stress components have been shown to be upregulated by IL-1α treatment and to further sensitise the cells to IL-1β treatment ^46– 49^.

Moreover, several ER stress markers, such as *ERN1* (encoding IRE-1), *PERK* and *CREB3L2* (encoding a transcription factor involved in the UPR), are decreased in OA chondrocytes ^48^. Whereas IL-1β treatment increases *ERN1* and *PERK* expression in OA chondrocytes, platelet-derived growth factor (PDGF-BB) and IL-6 stimulation leads to a decreased expression of both but does not affect the levels of ATF6β, which are also comparable between the healthy and OA chondrocytes. Finally, small interfering RNA (siRNA) silencing of *ERN1* led to decreased expression of *COL2A1*, *MMP-13*, and *ADAMTS-4* and *ADAMTS -5*, and silencing of *CREB3L2* showed a significant reduction of *ADAMTS-5*, which could be overcome by additional stimulation with IL-1β.

### An adapted endoplasmic reticulum stress response could delay the onset of osteoarthritis

A recent study by Kung *et al*. employed the dislocation of the medial meniscus (DMM) mouse model to study trauma-induced OA ^50^. Interestingly, BiP expression was markedly increased after DMM surgery in wild-type mice, clearly demonstrating an involvement of ER stress and the UPR in the pathogenesis of OA. However, whilst BiP levels were also increased upon DMM surgery in the transgenic mice overexpressing a misfolding variant of thyroglobulin (Tg ^cog^) under the *Col2a1* promoter (ColIITg ^cog^, a model of cartilage-specific ER stress ^51^), the onset of OA was delayed, and a delay in cartilage apoptosis and damage was seen two weeks after surgery compared with wild-type controls. Interestingly, ColIITg ^cog^ mice had elevated BiP protein levels prior to DMM surgery, and the pro-survival Xbp1-associated UPR signalling network was activated in ColIITg ^cog^ mice after DMM, suggesting that pre-exposure to ER stress or activation of the specific pro-survival UPR responses such as the *Xbp1s* signalling could be chondroprotective.

### ATF6α does not contribute to the capacity of the unfolded protein response in the context of osteoarthritis

Strikingly, although several studies suggested that ATF6 signalling may be responsible for the regulation of *Xbp1* during OA progression, no difference in OA onset or progression could be seen in ATF6α-deficient mice after DMM surgery, suggesting that ATF6α may not be as relevant in this context as other branches of the UPR ^50^. Previous studies have shown a differential expression of ATF6α and ATF6β in a cartilage growth plate and a differentiation state–dependent modulation of ATF6 signalling ^16^. Interestingly, silencing of *ATF6β* led to decreased *ADAMTS-5* and *ADAMTS-4* expression, indicating the importance of increasing our understanding of the UPR in the context of OA progression.

### PERK: a major factor in osteoarthritis?

CHOP is a pro-apoptotic transcription factor downstream of the PERK and ATF6 signalling during the UPR. CHOP levels are increased in OA cartilage ^52^ and in osteochondritis dissecans (OCD) cartilage degeneration in horses ^53^. It has been shown that CHOP can be upregulated as a result of inflammationor mechanical or oxidative stress in cartilage, all of which are important components of OA progression. Interestingly, chondrocyte apoptosis was decreased in CHOP null mice compared with wild-type controls, and OA-associated changes in expression of *Col2a1* and *Acan* were suppressed in the absence of CHOP ^42^. These data indicate that CHOP-mediated apoptosis may be an important component of OA progression.

A recent study by Hisanaga *et al*. investigated the role of PERK in ATDC5 cells and in primary chondrocytes from PERK-deficient mice ^54^. Interestingly, the absence of PERK led to a reduced secretion of collagen type II into the extracellular space in both models. Moreover, a decrease in PERK expression was detected in cultured OA chondrocytes, and siRNA silencing of *PERK* in chondrocytes led to a decrease in *COL2A1* expression (further aggravated by IL-1β treatment) and an increase in *COL1A1* expression. The switch from *COL2A1* to *COL1A1* expression is indicative of cartilage de-differentiation and fibrosis and is associated with later stages of OA ^55^; thus, these studies indicate that PERK modulation is a potential novel therapeutic target in OA.

### The unfolded protein response in osteoarthritis: one of many therapeutic targets

The UPR signalling pathway is part of the complex crosstalk of signals comprising cellular response to stress, which include inflammation and autophagy ^8, 56^. Autophagy is a process by which healthy chondrocytes remove their malfunctioning components and can be used to obtain energy during cellular stress, such as the UPR ^57^. Interestingly, pro-survival markers of autophagy microtubule-associated proteins 1A/1B light chain 3B (LC3) and beclin-1 are increased in early OA, but autophagy markedly decreases with OA severity ^53, 58, 59^. Moreover, deletion of mTOR from cartilage leads to an increase in autophagy and is chondroprotective ^60^.

Several pharmacological interventions in OA research also demonstrate the complexity of the crosstalk within the cellular stress pathways. Salubrinal (a chemical that elevates phosphorylation of eIF2α) is chondroprotective upon surgical induction of OA ^61^; however, guanabenz, which also increases phosphorylation of eIF2α, does not have that effect. Interestingly, salubrinal can also reduce the phosphorylation of NF-κB and modulate matrix metalloproteinase (MMP) signalling by influencing the inflammation signalling pathway. IL-1β can suppress anti-inflammatory 5′ AMP protein kinase (AMPK) signalling in OA chondrocytes, and silencing of AMPK leads to increased levels of CHOP. 5-Aminoimidazole-4-carboxamide ribonucleotide (AICAR) is an AMP kinase signalling agonist. Interestingly, AICAR treatment of biomechanically injured alginate cultured bovine chondrocytes reduces the levels of CHOP ^49^. GLP-1R (glucagon-like peptide-1 receptor involved in glucose and energy homeostasis) can regulate ER stress-induced apoptosis and inflammatory response during OA. GLP-1R agonist has been shown to be chondroprotective in an anterior cruciate ligament (ACL) surgery model of OA in rat and decrease the levels of CHOP and active caspase-3 in cartilage via modulation of phosphoinositide 3-kinase/protein kinase B/mTOR (PI3K/AKT/mTOR) signalling pathway that is involved in autophagy ^62^. H _2_O _2_ treatment of chondrocytes has been shown to trigger oxidative stress and to increase ER stress markers BiP, CHOP and caspase-12. Curcumin, an antioxidant and anti-inflammatory drug, can modulate the PERK/eIF2α/CHOP UPR axis through promoting sirtuin 1 (SIRT1) in chondrocytes and is chondroprotective in the ACL rat model of OA, leading to a decrease in CHOP and slower cartilage degradation ^41^. All of the above further emphasise the complex role of ER stress in OA progression and the need to dissect the crosstalk of the cellular stress pathways in disease onset and progression.

## What does the future hold?

May 2019 saw the first patients recruited to a multinational clinical trial looking to repurpose carbamazepine to treat metaphyseal chondrodysplasia type Schmid. This is the first time that ER stress has been targeted in a GSD, although ER stress is being treated in other conditions, including a range of rare diseases ^63^, diabetes and several cancers ^64^.

The ability to therapeutically target a ‘disease mechanism’, such as ER stress, at the cell and tissue level as opposed to correcting the primary genetic defect is a paradigm shift, particularly in the context of rare diseases. About 7000 different rare disease have been identified and described, but fewer than 400 have a licensed treatment. Historically, numerous challenges have been associated with developing and delivering therapies for rare diseases, including small and geographically dispersed patient populations, the properties of proposed therapies that are often highly (even genotype) specific, absence of a unified target and/or therapeutic approach and a limited market; all compromise commercial viability. Therefore, defining and targeting common disease mechanisms in groups of phenotypically unrelated diseases (‘common amongst the rare’), coupled with drug repurposing, offer an attractive route for accelerating translational rare disease research.

## Abbreviations

4-PBA, 4-phenylbutyric acid; ACL, anterior cruciate ligament; AICAR, 5-aminoimidazole-4-carboxamide ribonucleotide; AMPK, 5′ AMP protein kinase; ATF, activating transcription factor; BiP, binding immunoglobulin protein; CBZ, carbamazepine; CHOP, C/EBP homologous protein; COMP, cartilage oligomeric matrix protein; DMM, dislocation of the medial meniscus; ECM, extracellular matrix; eIF2α, eukaryotic translation initiation factor 2A; ER, endoplasmic reticulum; ERAD, endoplasmic reticulum–associated protein degradation; ERp57, endoplasmic reticulum resident protein 57; GLP-1R, glucagon-like peptide-1 receptor; GSD, genetic skeletal disease; IL, interleukin; IRE-1, inositol-requiring enzyme 1; ISRIB, integrated stress response inhibitor; MANF, mesencephalic astrocyte-derived neurotrophic factor; MCDS, metaphyseal chondrodysplasia type Schmid; MED, multiple epiphyseal dysplasia; MMP, matrix metalloproteinase; mTOR, mammalian target of rapamycin; NF-κB, nuclear factor kappa B; OA, osteoarthritis; PERK, protein kinase ribonucleic acid-like endoplasmic reticulum kinase; PSACH, pseudoachondroplasia; S1P, site 1 protease; UPR, unfolded protein response; XBP1, X-box binding protein 1.
